# Body mass index is not associated with survival outcomes and immune-related adverse events in patients with Hodgkin lymphoma treated with the immune checkpoint inhibitor nivolumab

**DOI:** 10.1186/s12967-021-03134-4

**Published:** 2021-12-01

**Authors:** Rosaria De Filippi, Fortunato Morabito, Armando Santoro, Giovanni Tripepi, Francesco D’Alò, Luigi Rigacci, Francesca Ricci, Emanuela Morelli, Pier Luigi Zinzani, Antonio Pinto

**Affiliations:** 1 Dipartimento di Medicina Clinica e Chirurgia, Università degli Studi Federico II, Napoli, Italy; 2 Ematologia Oncologica e Trapianto di Cellule Staminali, Department of Hematology and Developmental Therapeutics, Istituto Nazionale Tumori, Fondazione ‘G. Pascale’, IRCCS, Via Mariano Semmola 49, 80131 Naples, Italy; 3 Hemato-Oncology Department, Augusta Victoria Hospital, East Jerusalem, Israel; 4 Unità di Ricerca Biotecnologica, Azienda Ospedaliera di Cosenza, Cosenza, Italy; 5 Humanitas University, Milano, Italy; 6 IRCCS Humanitas Research Hospital, Milano, Italy; 7 Unità di Ricerca CNR-IFC, Reggio Calabria, Italy; 8 Dipartimento di Diagnostica per Immagini, Radioterapia Oncologica ed Ematologia, Fondazione Policlinico Universitario Agostino Gemelli IRCCS, Roma, Italy; 9 Dipartimento di Scienze Radiologiche ed Ematologiche, Università Cattolica del Sacro Cuore, Roma, Italy; 10 Ematologia e Trapianto di Cellule Staminali, A.O. San Camillo Forlanini, Roma, Italy; 11 IRCCS Azienda Ospedaliero-Universitaria di Bologna, Istituto di Ematologia “Seràgnoli”, Bologna, Italy

**Keywords:** Hodgkin lymphoma, Immune checkpoint inhibitors, Body mass index, Immune-related adverse events

## Abstract

**Background:**

Overweight and obese patients with solid tumors receiving anti-programmed cell death-1 (PD-1)/PD-ligand-1**(**PD-L1) immune checkpoint inhibitors exhibit improved survival and higher risk of immune-related adverse events (irAEs) than those with a normal body mass index (BMI). In classic Hodgkin lymphoma (cHL), the impact of BMI on survival and immune-related toxicity is unknown. We evaluated for the first time associations of BMI with survival and irAEs in patients with relapsed/refractory (RR)-cHL undergoing PD-1 blockade.

**Methods:**

Data from a multicenter study on 133 patients treated with the anti-PD1 antibody nivolumab (July 2015–December 2016) were retrieved from a prospective database. Progression-free (PFS), overall survival (OS), incidence and severity of irAEs according to BMI categories were estimated by Kaplan–Meier method, landmark-analyses and Cox regressions.

**Results:**

Patients, mostly males (63%, n = 84) with a median age of 35 years (range, 15–82), advanced stage (75%), B symptoms (63%), bulky disease (24%), a median of 4 previous treatments (range, 1–9), received a median of 18 nivolumab doses (range, 1–57). No statistically significant differences across BMI subgroups emerged as to PFS, with 1-year rates of 67.1% for both normal weight (n = 66; 49.6%) and overweight (n = 31; 23.3%) patients. Underweight (n = 12; 9%) and obese (n = 24; 18%) patients had a 1-year PFS of 54.5% and 49%, respectively. In survival analyses, BMI either as a continuous (P = 0.5) or categorical (P for trend = 0.63) variable failed to associate with PFS. Response rates and time-to-response did not cluster in any BMI subset. No BMI-related differences in OS emerged across normal, overweight and obese patients but underweight patients had the worst survival. Occurrence of irAEs of whatever severity did not statistically associate with BMI.

**Conclusions:**

In patients with RR-cHL receiving nivolumab, no statistically significant differences emerged in response rates, PFS and OS across BMI categories of normal weight, overweight and obese. Overweight/obese patients did not display an increased risk of irAEs. The exquisite sensitivity to anti-PD-1 antibodies, the unique cytokine milieu and effector pathways triggered by nivolumab in cHL, may represent biologic ‘equalizers’ counteracting the immunoregulatory effects of adiposity. Differently from solid tumors, BMI is not associated with treatment efficacy and immune-related toxicity and does not represent a predictive tool for PD-1-targeted immunotherapies in cHL.

**Supplementary Information:**

The online version contains supplementary material available at 10.1186/s12967-021-03134-4.

## Background

Studies across a variety of solid tumors have documented a statistically significant association between higher pretreatment body mass index (BMI) and improved survival outcomes in cancer patients receiving antibodies blocking the programmed cell death protein-1 (PD-1)/programmed death-ligand 1/2 (PD-L1/2) pathway [1]. It was shown that overweight patients display a longer progression-free survival (PFS) and overall survival (OS), compared with those of average weight or underweight [1–3].

Overweight/obese patients also show an increased risk of immune-related adverse events (irAEs), highlighting a mechanistic association between development of irAEs and improved clinical outcomes in the context of PD-1/PD-L1-targeted immunotherapies [4, 5]. Therefore, BMI has been proposed as a predictive tool in clinical practice and a stratification factor for trials of anti-PD-1/PD-L1 treatments [2, 5, 6].

The predictive value of BMI on survival and immune-related toxicity in classical Hodgkin lymphoma (cHL) is currently unknown. This is remarkable since cHL, also due to recurrent alterations of the 9p24.1 chromosomal region, which contains the PD-L1 and PD-L2 loci, is among the human malignancies most responsive to PD-1/PD-L1-targeted immunotherapies [7–9].

Here, we sought to investigate a possible association between BMI, clinical outcomes and occurrence of irAEs, on a study cohort of 133 patients with RR-cHL who received the anti-PD1 antibody nivolumab as a single agent. By doing so, we provide the first report on interactions between obesity, efficacy/survival outcomes and immune-related toxicity in the context of PD-1 blockade for cHL.

## Methods

### Patient selection and study procedures

A total of 140 patients with RR-cHL who received ≥ 1 dose of nivolumab monotherapy, according to a treatment protocol approved by local ethical committees at Italian Hematology-Oncology referral Centers, were registered between July 2015 and December 2016. Patients provided informed consent to treatment and data analysis for scientific purposes. Inclusion criteria and treatment details are given as Additional file 3. Nivolumab (3 mg/kg) was administered intravenously every 2 weeks until disease progression or unacceptable toxicity. Data were prospectively collected into a central database, verified, updated, and locked in December 2018 by three Study Coordinators (AS, PLZ, AP). After checking for inclusion criteria and full dataset availability, a validated database of 133 patients was utilized for all statistical evaluations (Additional file 1: Figure S1). Data extraction and analysis was performed within the INTHEMA study protocol (IRSTB100, L3P2065, NCT04298892), approved by local ethical committees of coordinating centers.

### Anthropometric measurements

For each patient, weight and height values were obtained at the time of nivolumab initiation and before administering the first dose. BMI was calculated according to weight/height^2^ (kilograms per square meter) formula, and patients were categorized by the WHO criteria as underweight, BMI < 18.5 kg/m^2^; normal-weight, 18.5 kg/m^2^ ≤ BMI ≤ 24.9 kg/m^2^; overweight, 25 kg/m^2^ ≤ BMI ≤ 29.9 kg/m^2^ and obese BMI ≥ 30 kg/m^2^.

### Efficacy and toxicity assessment

Complete response (CR), partial response (PR), stable disease (SD), and progressive disease (PD) were defined according to Lugano criteria [10]. AEs were graduated, according to Common Toxicity Criteria (CTCAE; version 4.0) and irAEs were defined upon multidisciplinary consultation and based on the following rank order list: (a) exclusion of alternative diagnoses; (b) clinico-pathologic and laboratory evidence of immunological nature; (c) clinical improvement upon nivolumab suspension and/or irAE-directed treatments. irAEs were graduated according to CTCAE v 4.0 and cumulatively reported [11]. AEs/irAEs leading to treatment discontinuation (LTD) were those causing a permanent interruption of nivolumab.

### Statistical methods and analyses

Data are expressed as absolute numbers and percentages. Statistical comparisons were performed using two-way tables for the Fisher’s exact test and multi-way tables for the Pearson’s Chi-square test for categorical variables. Mann–Whitney U test was utilized for the comparison between two groups of cases on the same variable. PFS and OS analyses were performed using the Kaplan–Meier method. Statistical significance of associations between individual variables and PFS or OS was calculated using the log-rank test. Univariate Cox regression analyses investigated the prognostic impact for the outcome variables. In the Cox models, data were expressed as hazard ratios (HR) and 95% confidence intervals (CI). A value of P < 0.05 was considered significant [12]. Cumulative PR or better (> PR) rates over time were evaluated from the start of nivolumab treatment. The landmark method was used to minimize the bias in favor of responders represented by the time required to reach the response [13]. Patients were classified as CR or < CR according to their best overall response at the landmark time. The choice of landmark time was based on the median time to reach a CR (3.7 months), to discontinue therapy because of AEs (1.4 months), and to undergo transplant (6.0 months). PFS events noted by landmark time were excluded from the analysis. All analyses were performed by SPSS for Windows Version 22, Chicago, Illinois, USA & STATA 13 for Windows StataCorp (Lakeway Drive, College Station, TX).

## Results

### Patient characteristics

A total of 133 patients were included in this study. As shown in Table 1, they were mostly males (63%, n = 84) with a median age of 35 years (range, 15–82), advanced stage (75%), B symptoms (63%), bulky disease (24%) and had received a median of 4 previous treatment lines (range: 1–9), including autologous stem cell transplantation (55%) or allogeneic stem cell transplantation (20%). The vast majority (96%) had also previously received Brentuximab vedotin (BV). Patients were administered a median of 18 nivolumab doses (range: 1–57).

**Table 1:** Baseline clinical features of 133 patients with RR-cHL treated with Nivolumab

Characteristics	No. (%)
No.	133
Age (years)	
Median, (range), years	35 (15–82)
Gender	
Male	84 (63.2)
Female	49 (36.8)
ECOG PS	
0–1	109 (81.9)
≥ 2	24 (18.1)
Histology	
Nodular sclerosing	106 (79.7)
Mixed cellularity	8 (6)
Lymphocyte rich	2 (1.5)
Lymphocyte depleted	2 (1.5)
Hodgkin lymphoma unspecified	15 (11.3)
B-symptoms	83/131 (63.3)
Bulky disease	31/131 (23.6)
Stage III–IV	98/130 (75.4)
Bone marrow involvement	12/109 (11)
Previous treatment lines	
Median (range)	4 (1–9)
1–2	8 (6)
3–4	68 (51.1)
≥ 5	57 (42.9)
Previous stem cell transplantation	
Autologous^a^	76 (57.1)
Allogeneic	28 (21)
Prior Brentuximab Vedotin	128 (96.2)
Nivolumab doses received	
Median (range)	18 (1–57)
BMI (kg/m^2^)	
Median (range)	24.1 (16.5–44.4)
Underweight (BMI ≤ 18.5)	12 (9)
Normal weight (BMI 18.5 < BMI ≤ 24.9)	66 (49.6)
Overweight (25 < BMI ≤ 29.9)	31 (23.3)
Obese (BMI ≥ 30)	24 (18.1)

*ECOG PS* Eastern Cooperative Oncology Group performance status, *BMI* body mass index according to WHO categorization

^a^Five patients received tandem autologous stem cell transplants

### Patients’ categorization according to BMI

Patient’s distribution across WHO BMI categories, at initiation of nivolumab, according to weight/height^2^ (kilograms/square meter) formula, is described in Table 1. The median BMI was 24.1 kg/m^2^; 12 patients (9%) were classified as underweight, 66 patients (49.6%) as having a normal weight, 31 patients (23.3%) as overweight, and 24 patients (18.1%) as obese, according to WHO criteria.

### Association of BMI with baseline clinical features

Disease-related baseline features before initiation of nivolumab, including presence of bulky disease, bone marrow involvement and types and number of previous treatments did not statistically cluster in any BMI category (Additional file 1: Figs. S2 and S3). Similarly, the presence of B symptoms did not statistically associate with any BMI subset, including underweight cases.

### Efficacy analysis according to BMI

**Fig. 1:**
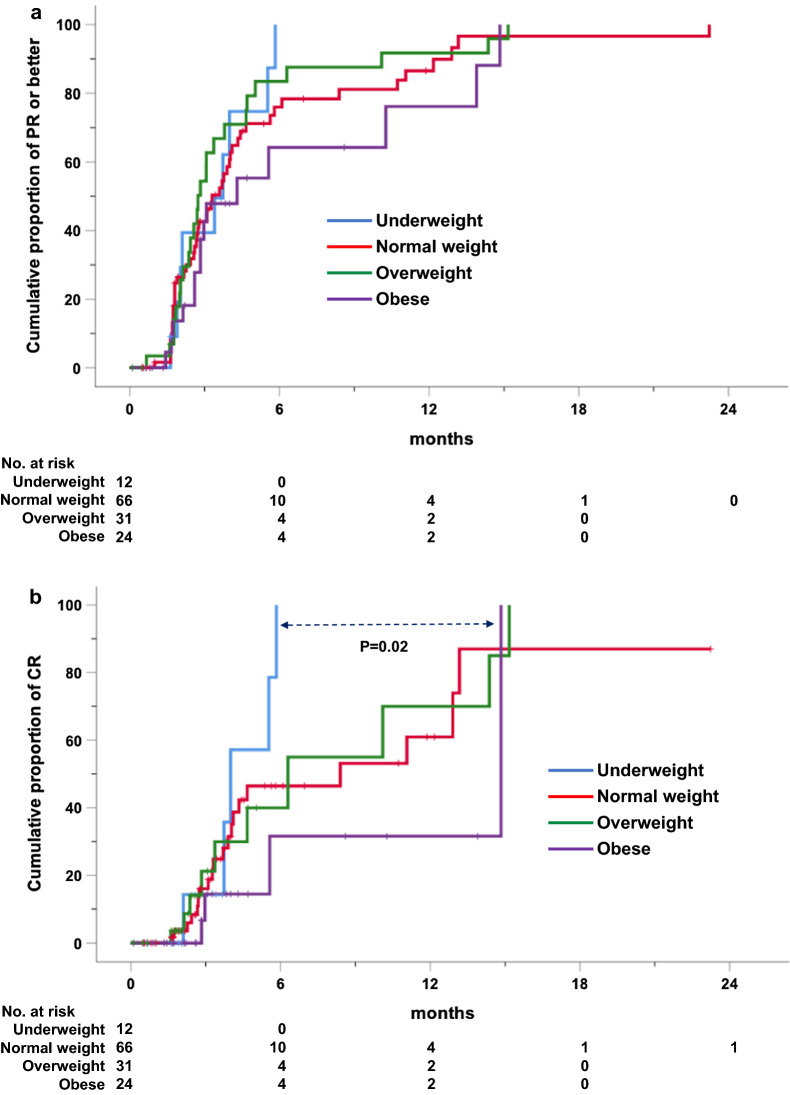
Cumulative proportion of partial responses or better (**a**) and complete responses (**b**) over time by body mass index categories. *PR* partial response, *CR* complete response

The overall response rate was 73.7% with 39 (29.3%) CRs, 59 (44.4%) PRs, 19 (14.3%) SD and 16 (12%) progressions. Best responses to Nivolumab evenly distributed across BMI categories (Table 2). Median times to ≥ PR and CR were of 3.3 and 6.3 months, respectively. Notably, achievement of ≥ PR was comparable among BMI subgroups (Fig. 1A), whereas time-to-CR favored underweight vs. obese patients (P = 0.02; Fig. 1B).

**Table 2:** Association between quality of response and body mass index clustered by standard WHO categories in patients with relapsed and refractory classical Hodgkin lymphoma treated with nivolumab monotherapy

		No. of responses (%)				
BMI	No. patients	CR	PR	SD	PD	*P* *
Underweight	12	4 (41.7)	4 (33.3)	1 (8.3)	2 (16.7)	
Normal weight	66	20 (30.3)	29 (43.9)	12 (18.2)	5 (7.6)	
Overweight	31	10 (32.9)	15 (48.4)	2 (6.5)	4 (12.9)	0.5
Obese	24	4 (16.7)	11 (45.8)	4 (16.7)	5 (20.8)	

*BMI* body mass index according to WHO categorization, *CR* complete response, *PR* partial response, *SD* stable disease, *PD* progressive disease

*Pearson Chi-Square

**Fig. 2:**
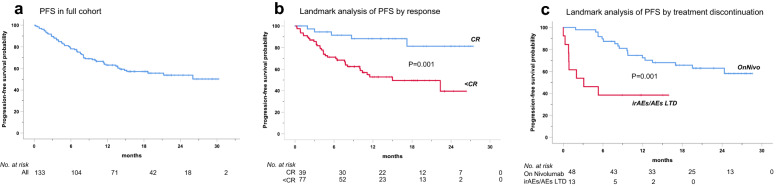
Kaplan–Meier estimates of PFS in patients with RR-cHL treated with anti-programmed Cell Death-1 monotherapy (nivolumab). PFS in the full study cohort (**a**), (**b**) landmark analysis of PFS by best response to nivolumab and (**c**) landmark analysis of PFS by treatment discontinuation due to toxicity compared with cases who remained on nivolumab therapy. The choice of landmark time was based on the median time to reach a CR, which was 3.7 months. PFS events noted by landmark time were excluded from the analysis. *CR* complete response, *< CR* partial responses and disease stabilization, *irAE* immune-related adverse event, *AE* non immune-related adverse event, *LTD* leading to treatment discontinuation, *OnNivo* patients who did not discontinued nivolumab

At 13.6 months of median follow-up (range: 1–30), 57 patients progressed or died; median PFS was not reached, with 1- and 2-year PFS rates of 63.1% and 53.8%, respectively (Fig. 2A; Table 3).

**Table 3:** One-year and 2-year rates and standard errors of progression free survival and overall survival by body mass index WHO categories in patients with relapsed and refractory classical Hodgkin lymphoma treated with nivolumab monotherapy

	PSF		OS	
	1-Year	2-Year	1-Year	2-Year
	% (± SE)	% (± SE)	% (± SE)	% (± SE)
All cases (n = 133)	63.1 (4.3)	53.8 (4.8)	87.6 (2.9)	81.2 (4.0)
BMI				
Underweight	54.5 (15.0)	54.5 (15.0)	75.0 (12.45)	60.0 (16.7)
Normal weight	67.1 (5.9)	53.6 (7.0)	90.6 (3.7)	85.3 (5.1)
Overweight	67.1 (8.6)	59.4 (9.2)	93.4 (4.5)	83.4 (8.1)
Obese	49.0 (10.4)	49.0 (10.4)	78.5 (8.5)	78.5 (8.5)

*PFS* progression free survival, *OS* overall survival, *SE* standard error, *BMI* body mass index according to WHO categorization

**Fig. 3:**
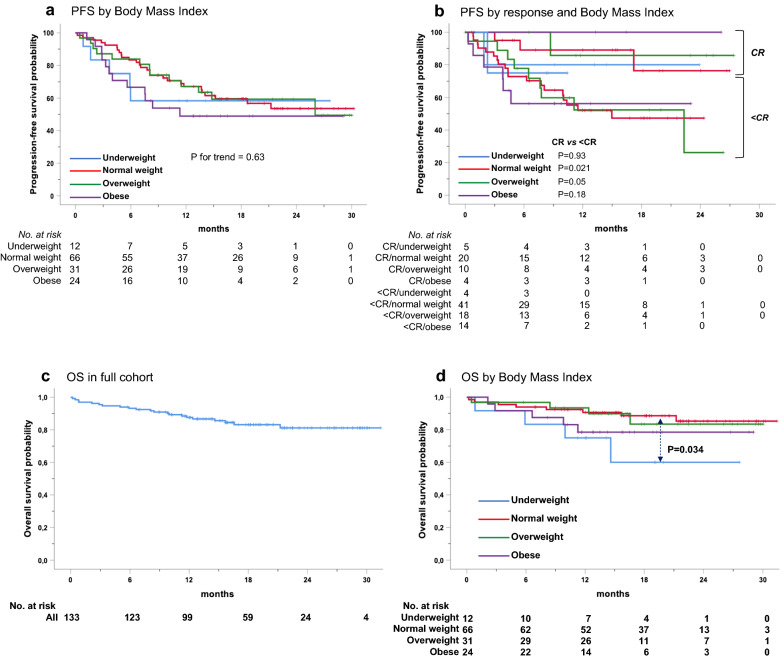
Kaplan–Meier survival estimates in patients with RR-cHL treated with anti-PD-1 monotherapy (nivolumab) according to BMI categories. PFS by BMI (**a**), landmark analysis of PFS by best response and BMI (**b**), OS in the full study cohort (**c**), OS by BMI (**d**). At a median follow-up of 16.4 months, median OS was not reached. Underweight patients had a significantly shorter OS as compared with those of normal weight (P = 0.027). *PD-1* programmed cell death-1, *RR-cHL* relapsed and refractory classic Hodgkin Lymphoma, *BMI* body mass index, *PFS* progression-free survival, *OS* overall survival

No disease-related variables were associated with response or PFS (Additional file 1: Figure S4) but a benefit emerged for CR vs. < CR patients (P < 0.001) (Fig. 2B). At last follow-up, 50 patients (37.6%) remained on treatment, 41 (30.8%) were bridged to transplantation, 16 (12%) discontinued nivolumab due to irAEs/AEs LTD, 22 (16.5%) progressed, one died and 3 (2 CRs, 1 PR), interrupted treatment due to physician choice. Landmark analyses for PFS demonstrated a significantly worse outcome for those patients who discontinued nivolumab because of irAEs/AEs LTD compared with those who did not (P = 0.001; Fig. 2C). Kaplan–Meier estimates did not evidence statistically significant PFS differences across BMI subgroups (Fig. 3A). One-year PFS rates for normal weight (n = 63, 27 events) and overweight (n = 31, 13 events) patients were 67.1% in both groups, while underweight (n = 12, 5 events) and obese patients (n = 24, 12 events), displayed the worse 1-year PFS of 58% and 49%, respectively (Table 3). BMI did not provide further risk stratification for CR vs. < CR patients (Fig. 3B).

**Fig. 4:**
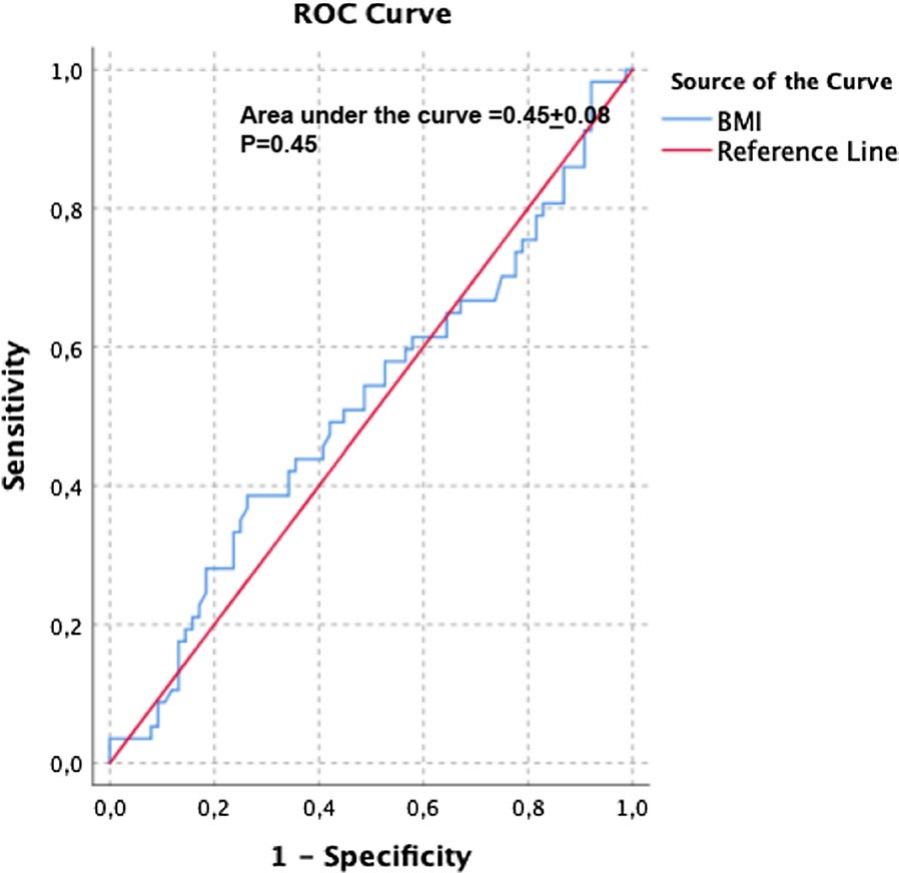
Receiver operating characteristic (ROC) analysis of body mass index (BMI) to identify patients who progressed during nivolumab monotherapy. The red line represents the reference line of predictive usefulness

Median OS was not reached, with 1- and 2-year OS rates of 87.6% and 86.7% (Fig. 3C; Table 3). No BMI-related differences in OS emerged across normal, overweight and obese patients; underweight patients had the worst survival (Fig. 3D). Finally, BMI failed to discriminate patients who progressed or died from those without these events, also in a specific receiver operating characteristics (ROC) analysis (Fig. 4).

### Association of irAEs and AEs with BMI categories

**Fig. 5:**
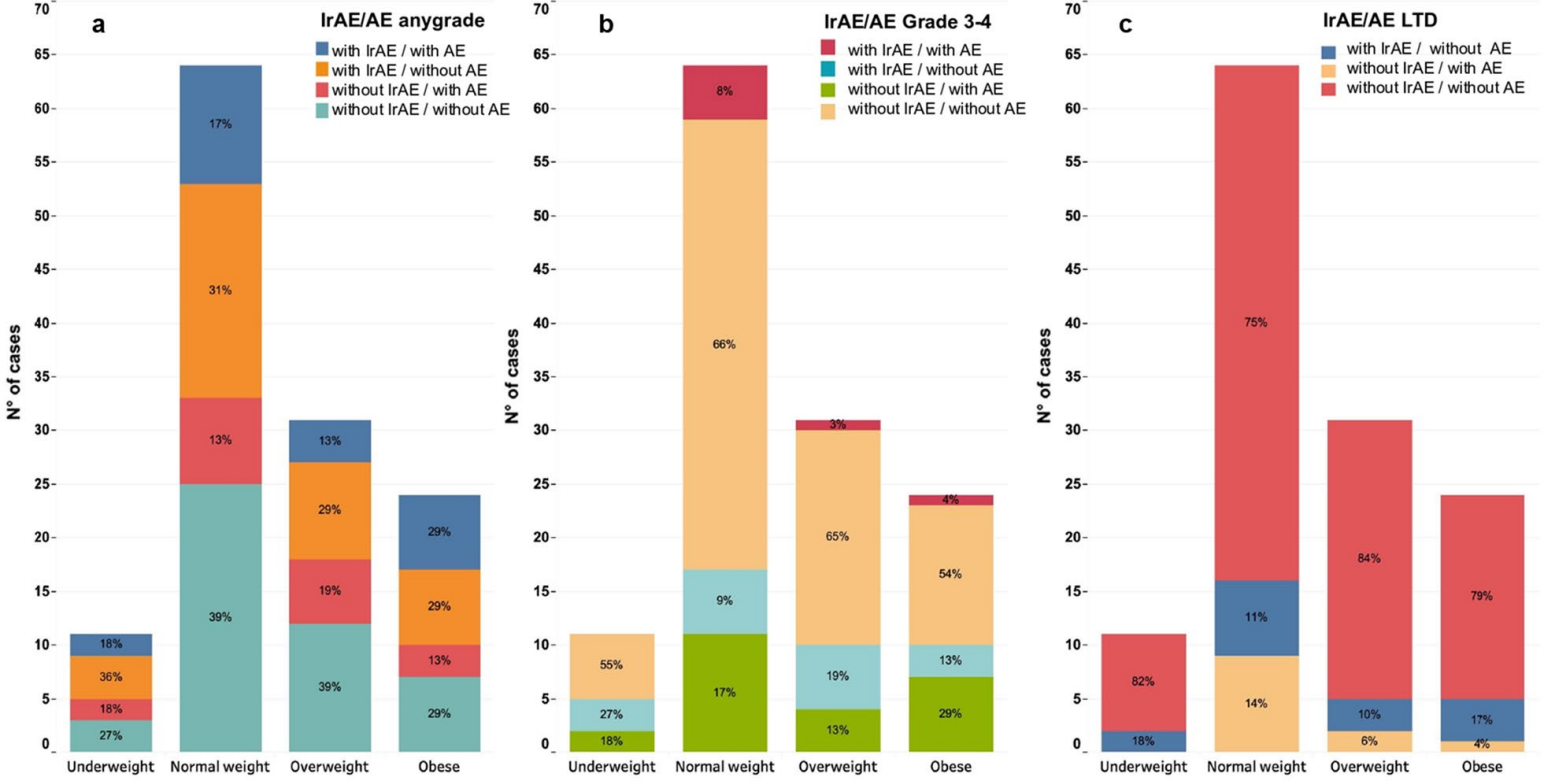
Occurrence of irAEs and AEs of any grade (**a**), of Grade 3–4 (**b**), and irAEs/AEs LTD (**c**) across the different body mass index categories. *irAE* immune-related adverse event, *AE* non immune-related adverse event, *LTD* leading to treatment discontinuation

Overall, 51.1% and 20% of patients experienced any grade and G3/G4 irAEs, respectively, while AEs of any grade and G3/G4 occurred in 31.8% and 15.9% of patients (Additional file 2: Table S1). Occurrence of AEs/irAEs of any grade, of G3-G4 severity and those LTD did not statistically cluster in any BMI subgroup (Table 4). Specifically, overweight and obese patients experienced irAEs of whatever severity, with or without concurrent AEs, at comparable frequencies and severities than normal weight or underweight patients (Fig. 5).

**Table 4:** Occurrence of irAEs and AEs of any grade, Grade 3/4 and leading to treatment discontinuation across body mass index categories according to the WHO classification

	BMI category				
Adverse events	Underweight	Normal weight	Overweight	Obese	P*
Any irAEs, n (%)	5 (41.7)	33 (50.8)	16 (51.6)	13 (56.5)	0.87
G3/G4 irAEs, n (%)	2 (16.7)	13 (20)	5 (16.1)	6 (27.3)	0.78
Any AEs, n (%)	4 (33.3)	17 (26.2)	10 (32.3)	11 (45.8)	0.37
G3/G4 AEs, n (%)	3 (25)	7 (10.8)	7 (22.6)	4 (16.7)	0.38
LDT irAEs, n (%)	2 (16.7)	3 (4.8)	2 (6.5)	4 (17.4)	0.20
LTD AEs, n (%)	0	3 (4.7)	2 (6.5)	0	0.54

*WHO* World Health Organization, *BMI* body mass index, *irAE* immune-related adverse event, *G* grade, *AE* immune non-related adverse event, *LTD* leading to treatment discontinuation

*Pearson Chi-Square

## Discussion

To our knowledge, this is the first study conducted in patients with cHL aimed at unveiling potential associations between BMI, incidence of irAEs, response outcomes and survival upon therapeutic PD-1 blockade with single agent nivolumab.

In our study any indicator of treatment efficacy, such as response rates and time-to-response, and survival outcomes did not associate with BMI categories that also evenly distributed in the context of known prognostic covariates for RR-cHL. Estimated PFS rates were comparable between patients with a normal weight and those who were underweight, overweight and/or obese. A specific ROC analysis documented that BMI categorization was unable to identify patients who progressed upon treatment with the anti-PD1 monotherapy. Furthermore, a landmark analysis of PFS according to the best response (CR vs. < CR), did not show BMI-related differences. Underweight patients had a shorter OS than those with normal weight, consistent with reports in solid tumors [2, 3]. Similarly, no relationships emerged between overweight/obesity and increased risk of irAEs of whatever severity. These negative results were confirmed by pooling obese/overweight *vs.* normal weight patients, using a binomial cut-off (BMI < / ≥ 25) and upon a separate re-analysis according to sex and creatinine values (< 0.9 mg/dL) to account for sarcopenic obesity [14, 15]. Regardless of BMI, landmark analyses evidenced instead that drivers such as quality of best response and risk of treatment discontinuation were strong outcome predictors.

The reasons underlying the lack of predictive capacity of BMI in patients with RR-cHL receiving PD-1 blockade can be multifaceted. While other neoplasms express variable amounts of PD-L1/2, tumor cells of cHL display a high density of PD-L1/2 in more than 98% of cases, due to a typical genetic alteration involving 9q24.1 [7–9]. The resulting exquisite sensitivity of cHL to PD-1 blockade, the highest among all tumors, might represent a biological ‘equalizer’ minimizing the predictive power of BMI. Interestingly, however, while it was suggested that in patients with non-small cell lung cancer given first line anti-PD-1 antibodies, the predictive effect of BMI on PFS and OS could be lost if tumor cells strongly express (≥ 50%) PD-L1, other studies confirmed that baseline obesity is associated to significantly improved clinical outcomes also in strong PD-L1 expressors [2, 3, 16, 17].

From a different perspective, it is known that adipose tissue regulates antitumor responses [1, 18]. Obesity-related meta-inflammation leads to tissue and systemic overproduction of cytokines/chemokines that induce tumor microenvironment remodeling, T-cell dysfunctions and exhaustion of cytotoxic CD8 + T-cells [1, 18]. cHL is a prototypical cytokine-overproducing tumor and patients display elevated amounts of circulating cytokines/chemokines, including those involved in adiposity-related immunoregulation [19]. These abnormally high cytokine levels are found both at presentation or relapse, and are associated with the occurrence of B symptoms, which may include weight loss [20]. Interestingly, the finding that in renal cell carcinoma, obesity is associated with a diminished efficacy of PD-1 blockade, has been ascribed, at least in part, to the presence of constitutively elevated IL-1β levels which can also frequently found in the microenvironment of cHL [21, 22]. This might have blunted the immunoregulatory effect of adipose tissues on outcomes of anti-PD-1 treatments. Notably, we were unable to show any statistically significant association of BMI categories with baseline clinical features, including B symptoms. More significantly, CD8 + T-cells are scarcely present in cHL microenvironment and restoration of cytotoxic T-cells does not represent a major determinant for PD-1 blockade efficacy in this lymphoma that typically lack MHC class I [8, 23]. Thus, if PD-1 blockade in solid tumors reverses a obesity-related impairment of cytotoxic CD8 + T cells functioning, this mechanism could not apply to cHL [23, 24].

Finally, while several studies in the context of cancer immunotherapy with PD-1/PD-L1 immune checkpoint inhibitors have demonstrated the favorable impact of obesity, other authors have highlighted that BMI itself may not always represent a most valid surrogate for body composition [25]. Under this light it has been shown that analysis of other parameters including skeletal muscle index and density or total adipose tissue is needed to better capture the functional interface of body composition on endogenous anti-tumor response and efficacy of PD-1 blockade [25].

Our study certainly suffers from limitations mainly due to its retrospective nature, data collection biases and lack of a centralized response assessment. It is of note however that inclusion criteria, patients characteristics, overall efficacy and toxicity outcomes of the present cohort, strikingly overlap with those from registrative studies of nivolumab in the setting of RR-cHL [7]. Nevertheless, unavoidable biases may restrain generalizability of present results.

## Conclusions

Our data indicate that BMI is not associated with survival outcomes and risk of irAEs in patients with RR-cHL treated with anti-PD-1 monotherapy. The association of adiposity with survival and immunologic toxicity in cancer patients treated with anti-PD-1/PD-L1 agents remains extremely complex also due to some conflicting results, negative reports and, above all, to a still inadequate understanding of its clinico-pathologic and immunologic determinants [2, 3, 14, 16, 21, 24, 25]. Under this light our results may stimulate further research in the specific field of cHL, including assessment patients who receive PD-1 blockade upfront and/or in earlier treatment lines, to clarify why the 'obesity paradox' does not seem to apply to this tumor that is other way exquisitely sensitive to PD-1 blockade.

## Supplementary Information

**Additional file 1: Fig. S1.** Study design, included and excluded patients. **Fig. S2.** Relationship between Body Mass Index (BMI) categories and potentially unfavorable disease-related baseline features. **Fig. S3.** Relationship between Body Mass Index (BMI) categories and types and number of previous therapies. **Fig. S4.** Forest plot of Cox univariate analysis for progression-free survival according to several variables. ASCT, autologous stem cell transplant, Allo-SCT, allogeneic stem cell transplant transplant.

**Additional file 2: Table S1.** Cumulative incidence of immune-related and non-immune related adverse events of any grade, of grade 3–4 and of those leading to treatment discontinuation in patients with relapsed and refractory classical Hodgkin lymphoma treated with nivolumab monotherapy.

**Additional file 3.** Inclusion criteria and participating centers and investigators.

## Data Availability

Deidentified patient dataset is available from the corresponding author on reasonable request.

## Abbreviations

PD-1: Programmed cell death-1
PD-L1: Programmed cell death-ligand-1
irAE: Immune-related adverse event
BMI: Body mass index
cHL: Classic Hodgkin Lymphoma
RR: Relapsed and refractory
PFS: Progression-free survival
OS: Overall survival
PD-L2: Programmed cell death-ligand-2
WHO: World health organization
CR: Complete responses
PR: Partial response
SD: Stable disease
PD: Progressive disease
AE: Non immune-related adverse event
CTCAE: Common toxicity criteria
LTD: Leading to treatment discontinuation
BV: Brentuximab vedotin
G: Grade
IL-1β: Interleukin-1β
MHC: Major histocompatibility complex
ROC: Receiver operating characteristic
