# Supplementary material for: Body mass index is not associated with survival outcomes and immune-related adverse events in patients with Hodgkin lymphoma treated with the immune checkpoint inhibitor nivolumab
Source: J Transl Med. 2021 Dec 1;19:489. doi: 10.1186/s12967-021-03134-4 (PMC8638339; doi:10.1186/s12967-021-03134-4)
Supplement: Supplementary file 1 — Additional file 1: Fig. S1. Study design, included and excluded patients. Fig. S2. Relationship between Body Mass Index (BMI) categories and potentially unfavorable disease-related baseline features. Fig. S3. Relationship between Body Mass Index (BMI) categories and types and number of previous therapies. Fig. S4. Forest plot of Cox univariate analysis for progression-free survival according to several variables. ASCT, autologous stem cell transplant, Allo-SCT, allogeneic stem cell transplant transplant. [file 12967_2021_3134_MOESM1_ESM.pptx]

## Slide 1
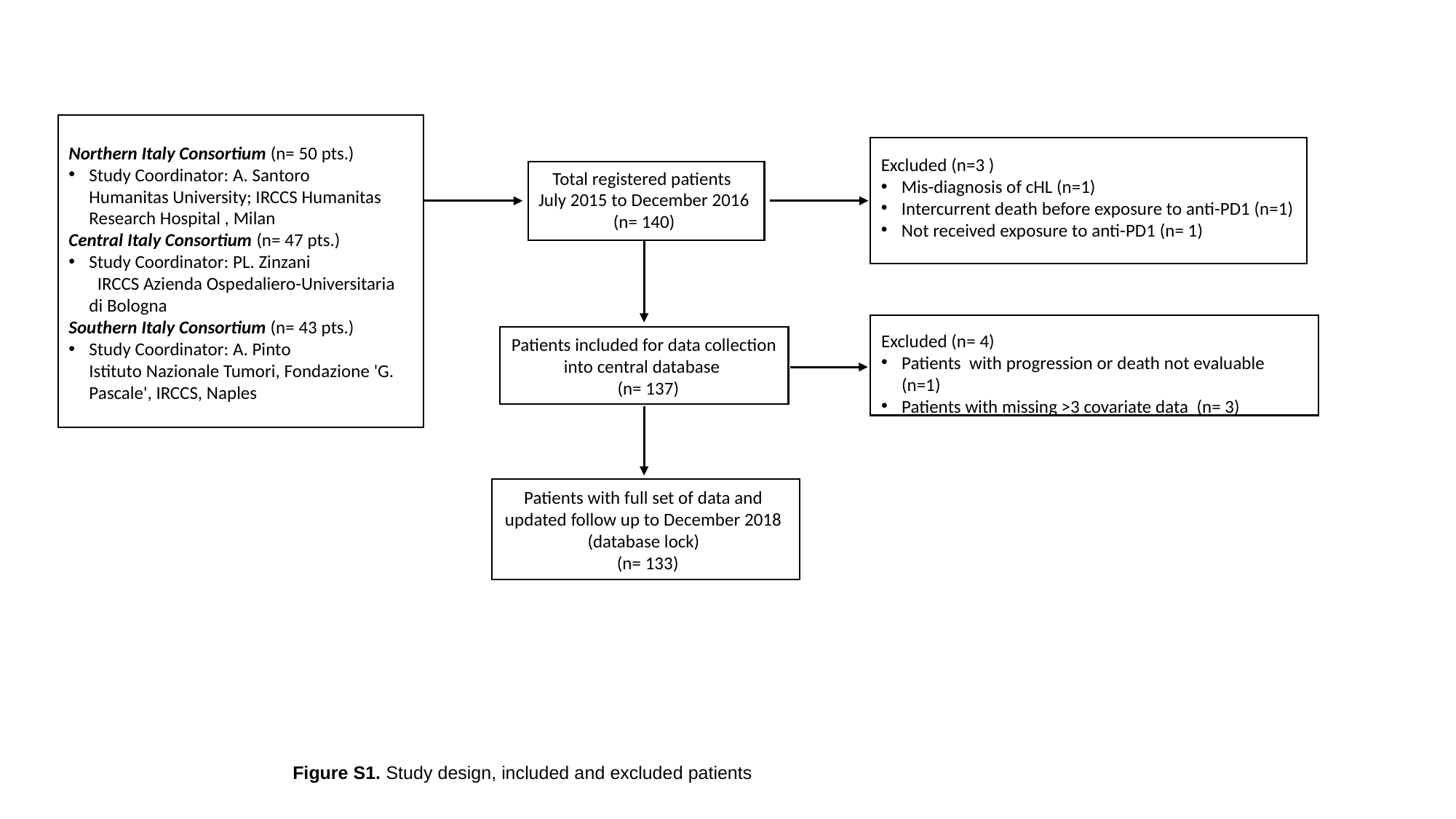

Northern Italy Consortium (n= 50 pts.)
Study Coordinator: A. Santoro Humanitas University; IRCCS Humanitas Research Hospital , Milan
Central Italy Consortium (n= 47 pts.)
Study Coordinator: PL. Zinzani IRCCS Azienda Ospedaliero-Universitaria di Bologna
Southern Italy Consortium (n= 43 pts.)
Study Coordinator: A. Pinto Istituto Nazionale Tumori, Fondazione 'G. Pascale', IRCCS, Naples
Excluded (n=3 )
Mis-diagnosis of cHL (n=1)
Intercurrent death before exposure to anti-PD1 (n=1)
Not received exposure to anti-PD1 (n= 1)
Total registered patients
July 2015 to December 2016
(n= 140)
Excluded (n= 4)
Patients with progression or death not evaluable (n=1)
Patients with missing >3 covariate data (n= 3)
Patients included for data collection into central database
 (n= 137)
Patients with full set of data and updated follow up to December 2018 (database lock)
 (n= 133)
Figure S1. Study design, included and excluded patients

## Slide 2
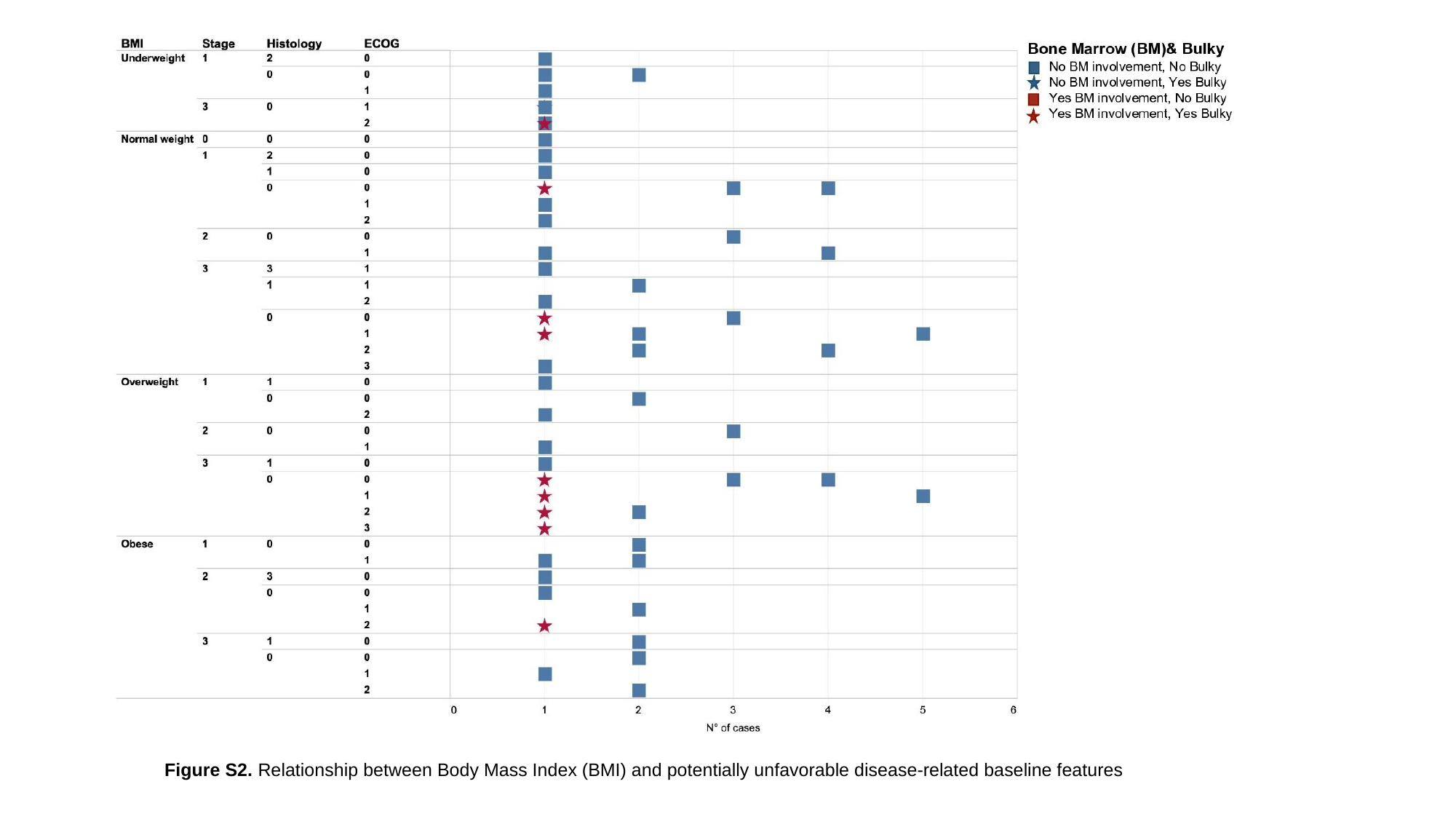

Figure S2. Relationship between Body Mass Index (BMI) and potentially unfavorable disease-related baseline features

## Slide 3
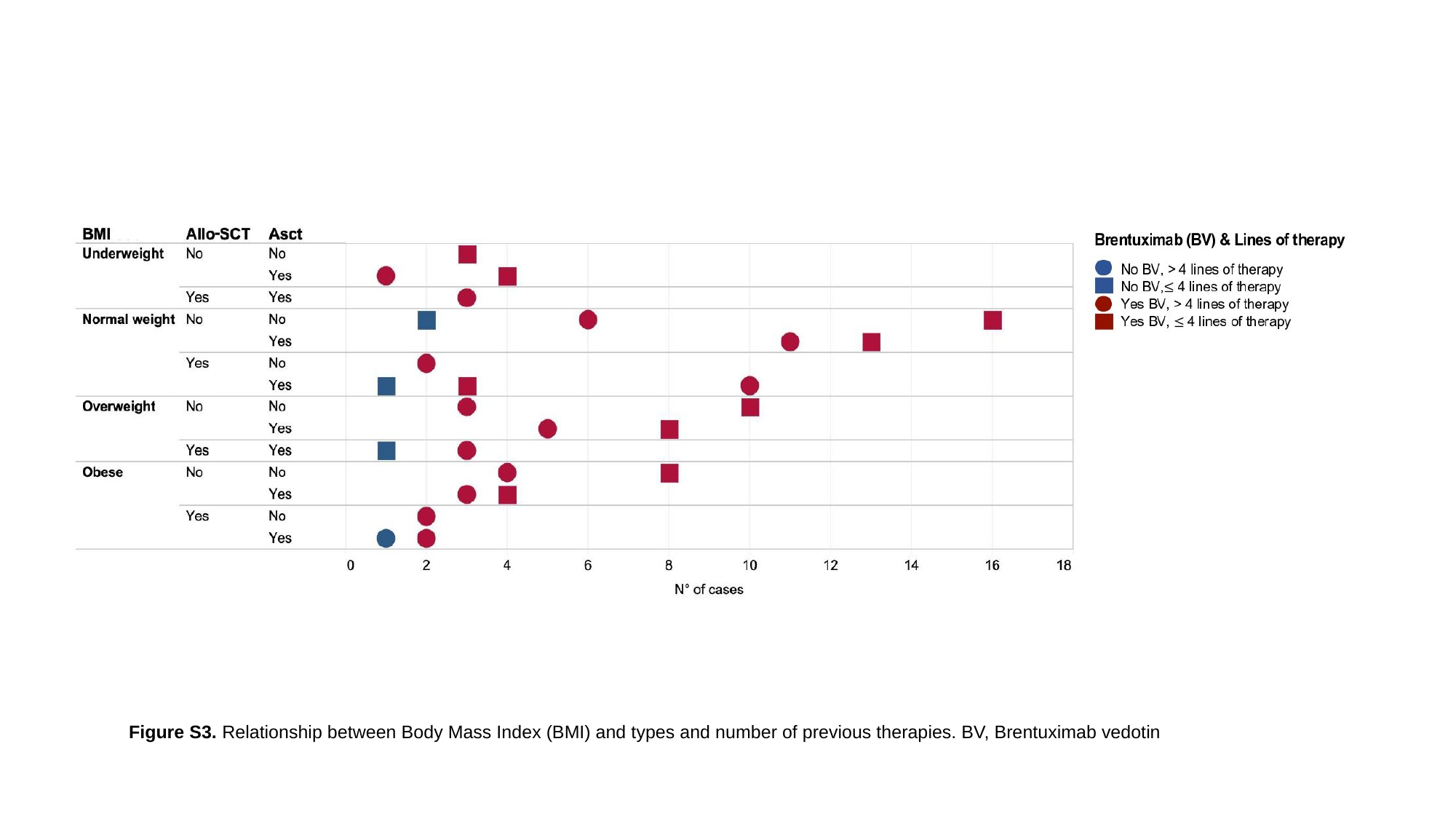

Figure S3. Relationship between Body Mass Index (BMI) and types and number of previous therapies. BV, Brentuximab vedotin

## Slide 4
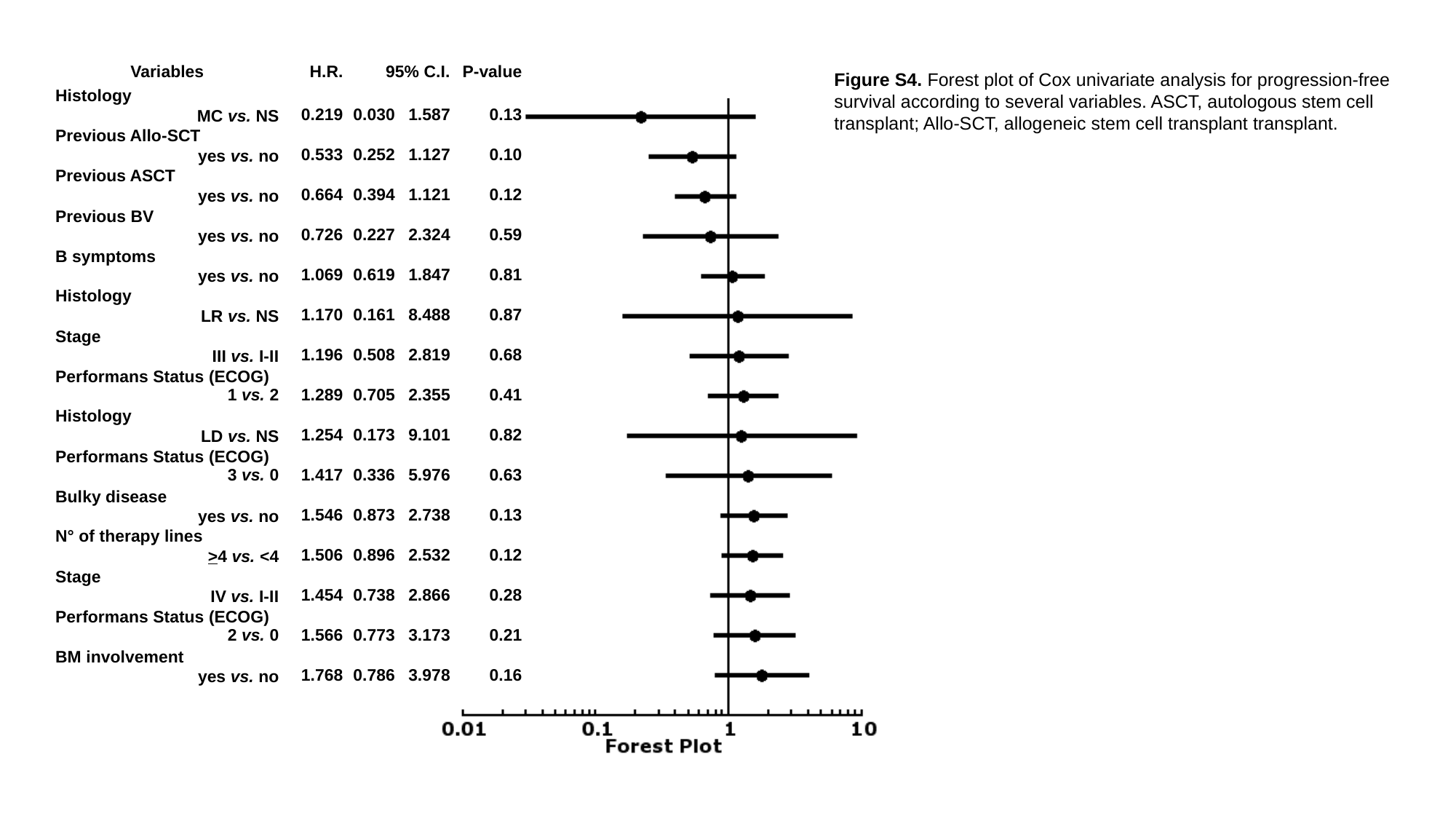

| Variables | H.R. | 95% C.I. | | P-value |
| --- | --- | --- | --- | --- |
| Histology | 0.219 | 0.030 | 1.587 | 0.13 |
| MC vs. NS | | | | |
| Previous Allo-SCT | 0.533 | 0.252 | 1.127 | 0.10 |
| yes vs. no | | | | |
| Previous ASCT | 0.664 | 0.394 | 1.121 | 0.12 |
| yes vs. no | | | | |
| Previous BV | 0.726 | 0.227 | 2.324 | 0.59 |
| yes vs. no | | | | |
| B symptoms | 1.069 | 0.619 | 1.847 | 0.81 |
| yes vs. no | | | | |
| Histology | 1.170 | 0.161 | 8.488 | 0.87 |
| LR vs. NS | | | | |
| Stage | 1.196 | 0.508 | 2.819 | 0.68 |
| III vs. I-II | | | | |
| Performans Status (ECOG) 1 vs. 2 | 1.289 | 0.705 | 2.355 | 0.41 |
| Histology | 1.254 | 0.173 | 9.101 | 0.82 |
| LD vs. NS | | | | |
| Performans Status (ECOG) 3 vs. 0 | 1.417 | 0.336 | 5.976 | 0.63 |
| Bulky disease | 1.546 | 0.873 | 2.738 | 0.13 |
| yes vs. no | | | | |
| N° of therapy lines | 1.506 | 0.896 | 2.532 | 0.12 |
| >4 vs. <4 | | | | |
| Stage | 1.454 | 0.738 | 2.866 | 0.28 |
| IV vs. I-II | | | | |
| Performans Status (ECOG) 2 vs. 0 | 1.566 | 0.773 | 3.173 | 0.21 |
| BM involvement | 1.768 | 0.786 | 3.978 | 0.16 |
| yes vs. no | | | | |
Figure S4. Forest plot of Cox univariate analysis for progression-free survival according to several variables. ASCT, autologous stem cell transplant; Allo-SCT, allogeneic stem cell transplant transplant.
