# Supplementary material for: Body mass index is not associated with survival outcomes and immune-related adverse events in patients with Hodgkin lymphoma treated with the immune checkpoint inhibitor nivolumab
Source: J Transl Med. 2021 Dec 1;19:489. doi: 10.1186/s12967-021-03134-4 (PMC8638339; doi:10.1186/s12967-021-03134-4)
Supplement: Supplementary file 2 — Additional file 2: Table S1. Cumulative incidence of immune-related and non-immune related adverse events of any grade, of grade 3–4 and of those leading to treatment discontinuation in patients with relapsed and refractory classical Hodgkin lymphoma treated with nivolumab monotherapy. [file 12967_2021_3134_MOESM2_ESM.pdf]

**Table S1.** Cumulative incidence of immune-related and non-immune related adverse events of any grade, of grade 3-4 and of those leading to treatment discontinuation in patients with relapsed and refractory classical Hodgkin Lymphoma treated with nivolumab monotherapy.

| **Adverse Events** | | **Number of Cases (%)** | |
|---|---|---|---|
| **irAEs** | **Any Grade** | **Grade 3 - 4** | **LTD** |
| No | 64 (48.9) | 104 (80.0) | 118 (91.5) |
| Yes | 67 (51.1) | 26 (20.0) | 11 (8.5) |
| Unknown | 2 | 3 | 4 |
| **AEs** | | | |
| No | 90 (68.2) | 111 (84.1) | 126 (96.2) |
| Yes | 42 (31.8) | 21 (15.9) | 5 (3.8) |
| Unknown | 1 | 1 | 2 |

irAEs: immune-related adverse events; AEs: non-ir adverse events; LTD: leading to treatment discontinuation
