# Supplementary material for: Body mass index is not associated with survival outcomes and immune-related adverse events in patients with Hodgkin lymphoma treated with the immune checkpoint inhibitor nivolumab
Source: J Transl Med. 2021 Dec 1;19:489. doi: 10.1186/s12967-021-03134-4 (PMC8638339; doi:10.1186/s12967-021-03134-4)
Supplement: Supplementary file 3 — Additional file 3. Inclusion criteria and participating centers and investigators. [file 12967_2021_3134_MOESM3_ESM.pdf]

De Filippi et al.

Additional File 3. Study details, inclusion criteria and participating institutions and investigators

Study population

This was an observational multicentre study aimed at describing the characteristics and outcome of patients with relapsed and refractory (RR) classical Hodgkin Lymphoma (HL), treated with single agent Nivolumab from July 2015 to November 2016, in Italian Hematology-Oncology referral Centres. Patients provided informed consent to data collection and use for scientific purposes. Data on patient characteristics and outcomes were extracted from local medical records and forwarded to Study Coordinators in electronic format. Centers providing data were grouped into three consortia according to the geographic location and patients were registered after revision of the inclusion criteria by three Study Coordinators: Northern Italy Consortium (n= 50 patients), Coordinator Prof. A. Santoro (Humanitas University, IRCCS Humanitas Research Hospital, Milan); Central Italy Consortium (n= 47 pts.), Coordinator Prof. PL. Zinzani (IRCCS Azienda Ospedaliero-Universitaria di Bologna); Southern Italy Consortium (n= 43 pts.), Coordinator Dr A. Pinto (Istituto Nazionale Tumori, Fondazione 'G. Pascale', IRCCS, Naples).

Inclusion criteria

Patients, were included if they were aged ≥18 years, had a histologically confirmed diagnosis of cHL, signed informed consent and received ≥ 1 dose of the anti-PD-1 antibody Nivolumab. Disease-related entry criteria were as follows: patients who failed or were unsuitable for autologous stem cell transplantation (SCT), patients who failed brentuximab vedotin) BV or had a specific contraindication to the agent. Patients with prior allogeneic SCT who fulfil the above criteria and all of the following criteria: a) Six or more months had elapsed since allogeneic SCT; b) no history of acute GVHD; c) no history of extensive or Grade 4 chronic graft vs. host disease (GVHD); d) free from immunosuppressive therapy for a minimum of 4 weeks and without clinically apparent GVHD. Nivolumab was administered at the dosage of 3 mg/kg body weight, IV every 2 weeks to a maximum of 24 months or until unacceptable toxicity, disease progression, or withdrawal of informed consent. A longitudinal survey was carried out by collecting data of patients who received at least 1 dose of Nivolumab.

Data Handling and analysis

From July 2015 to December 2016, data from a total of 140 patients were transferred to Study Coordinators for shared eligibility screen (Figure S1). Three patients were excluded upfront and data from 137 patients were

included into a shared central database. Four patients were further excluded by Study Coordinators based on the lack of covariate data. The database was updated and verified, through queries to local investigators, and locked in December 2018 by the Study Coordinators. The locked database was utilized for all statistical evaluations.

Partecipating Centres and investigators.

Norther Italy Consortium: R. Bassan, MD (ULSS12 Veneziana, Mestre), R. Cairoli, MD (ASST Grande Ospedale Metropolitano Niguarda, Milano), F. Ciceri, MD (Ospedale S. Raffele, Milano), P. Corradini, MD (Istituto nazionale Tumori, Milano), R. Fanin (AOU S. Maria Misericordia Udine), M. Gobbi, MD (Ospedale San Martino, Genova), D. Rapezzi, MD (AO Santa Croce e Carle, Cuneo), G. Rossi, MD, D.Russo, MD (Spedali Civili, Brescia), F.G. Rossi, MD (Policlinico, Milano), G. Semenzato, MD (AOU Padova), C Tarella, MD (Istituto Europeo di Oncologia, Milano). D. Vallisa, MD (Ospedale da Saliceto, Vicenza), U. Vitolo, MD (Città della Salute e della Scienza, Torino).

Central Italy Consortium: E. Abbruzzese, MD (Ospedale S. eugenio, Roma), F. Angrilli, MD (Ospedale di Pescara), A. Cantonetti, MD (Policlinico Tor Vergata, Roma), A. Cuneo, MD (AOU Ferrara, Arcispedale), A. Fabbri, MD (Azienda Ospedaliera Universitaria Senese),

B. Falini, MD (AOU Perugia), P. Galieni, MD (Ospedale Mazzoni, Ascoli Piceno), F. Lanza, MD (Ospedale S. Maria delle Croci, Ravenna), F. Merli, MD (Ospedale S.M. Nuova, Reggio Emilia), A. Pession, MD (Policinico S. Orsola Malpighi, Bololgna), P. Tosi, MD (AO di Rimini).

Southern Italy Consortium: G. Cabras, MD (AO A. Businco, Cagliari), C. Califano, MD (PO Tortora, Pagani), N. Cascavilla, MD (IRCCS Casa Sollievo della Sofferenza, S.G Rotondo), U. Consoli, MD (AO Garibaldi-Nesina Catania), N. Di Renzo, MD (PO Vito Fazzi, Lecce), C. Patti, MD (AOOR Villa Sofia-Cervello, Palermo), F. Ferrara, MD (AORN Cardarelli, Napoli), G. La Nasa, MD (PO Binaghi, Cagliari), D. Mannina, MD (Ospedale Papardo, Messina), S. Molica, MD (AO Pugliese-Ciaccio, Catanzaro), M. Musso, MD (La Maddalena, Palermo)
